# Homozygous Ala65Pro Mutation with V89L Polymorphism in SRD5A2 Deficiency

**DOI:** 10.4274/jcrpe.2495

**Published:** 2016-06-06

**Authors:** Erdal Eren, Tuba Edgünlü, Emre Asut, Sevim Karakaş Çelik

**Affiliations:** 1 Harran University Faculty of Medicine, Department of Pediatric Endocrinology, Şanlıurfa, Turkey; 2 Sıtkı Koçman University Faculty of Health Sciences, Department of Medical Biology, Muğla, Turkey; 3 Uludağ University Faculty of Medicine, Department of Pediatrics, Bursa, Turkey; 4 Bülent Ecevit University Faculty of Medicine, Department of Medical Genetics, Zonguldak, Turkey

**Keywords:** 46, XY disorders of sex development, 5-alpha-reductase, testosterone, mutation, polymorphism

## Abstract

**Objective:**

Deficiency of steroid 5-alpha reductase type 2 (5αRD2) is a rare autosomal recessive disorder caused by mutations in the SRD5A2 gene. A defect in the 5-alpha reductase enzyme, which ensures conversion of testosterone into dihydrotestosterone, leads to disorders of sex development. This study presents the clinical and genetic results of patients with 5αRD2 deficiency.

**Methods:**

5αRD2 deficiency was detected in 6 different patients from 3 unrelated families. All patients were reared as girls. Two of the patients presented with primary amenorrhea, one with primary amenorrhea and rejection of female gender, and the others with masses in their inguinal canals. Chromosome and sex-determining region Y (SRY) gene analyses were performed in all patients. Additionally, five exons of the SRD5A2 gene were amplified with polymerase chain reaction in the obtained DNA samples and evaluated.

**Results:**

While 46,XY was identified in 5 patients, 47,XXY was detected in one patient. The SRY gene was positive in all patients. The p.Ala65Pro (c193G>C) mutation and V89L polymorphism were observed in exon 1 of the SRD5A2 gene in all patients.

**Conclusion:**

Identification of this mutation and polymorphism is a significant indicator of presence of 5αRD2 deficiency in Southeastern Turkey, a geographical region where consanguineous marriages are also highly common.

**WHAT IS ALREADY KNOWN ON THIS TOPIC?**

5-alpha reductase type 2 deficiency can cause disorders of sex development. p.Ala65Pro (c193G>C) mutation has been reported before in Turkey.

**WHAT THIS STUDY ADDS?**

The p.Ala65Pro (c193G>C) mutation with V89L polymorphism is reported first time. This association can be found in Southeast region of Turkey.

## INTRODUCTION

Steroid 5-alpha reductase type 2 (5αRD2) deficiency is a rare autosomal recessive disorder caused by mutations in the SRD5A2 gene. A defect in the 5-alpha reductase enzyme, which ensures conversion of testosterone (T) into dihydrotestosterone (DHT), leads to disorders of sex development (DSD) (1). The 5-alpha reductase enzyme has two isoenzymes (namely, SRD5A1 and SRD5A2), and SRD5A2 is found in genital skin tissue. Depending on the level of enzyme deficiency caused by the changes in this gene, patients may develop various phenotypic characteristics, including perineal hypospadias, bifid scrotum, micropenis, and complete female phenotype (2). It has been indicated that enzyme activity is affected by the polymorphisms in the SRD5A2 gene. The most commonly known of these are the TA repeat polymorphism in the 3’-untranslated region and the polymorphisms ensuring mutation of tyrosine to alanine in codon 49’, as well as the mutation of alanine to leucine codon 89 (3).

This study presents six patients who were diagnosed with 5αRD2 deficiency and showed mutation and polymorphism association.

## METHODS

### Patient Studies

#### Patient 1, Family 1

Patient 1, a 12-year-old patient who presented to our endocrine clinic with complaints of amenorrhea and gender dysphoria had not been examined by another physician before. There was a consanguineous marriage between the parents. The patient’s height was 176.4 cm and she weighed 63.3 kg. Thelarche was stage 1 and pubarche was stage 4 according to Tanner’s classification. Gonads were palpable in both inguinal canals, and genital ambiguity was stage 3 according to Sinnecker’s classification. The two masses, 10x24 mm and 15x25 mm, visualized in right and left inguinal canals in a pelvic magnetic resonance imaging (MRI), were consistent with testicles. Laboratory findings were as follows: follicle-stimulating hormone (FSH) 4.97 IU/L, luteinizing hormone (LH) 4.26 IU/L, total T 2350 pg/mL, and DHT 70 pg/mL. Serum T to DHT ratio was found to be 33.5, and genetic evaluation was performed regarding 5αRD2 deficiency. Examinations revealed a 46,XY karyotype and a positive sex-determining region Y (SRY). Additionally, the patient was found to a have a homozygous mutation of p.Ala65Pro (c193G>C) in exon 1 of the SRD5A2 gene, as well as a p.Leu89Val (V89L) polymorphism. The patient was asked to come back for a follow-up evaluation regarding gonadectomy.

#### Patient 2, Family 2

**Figure 1:**
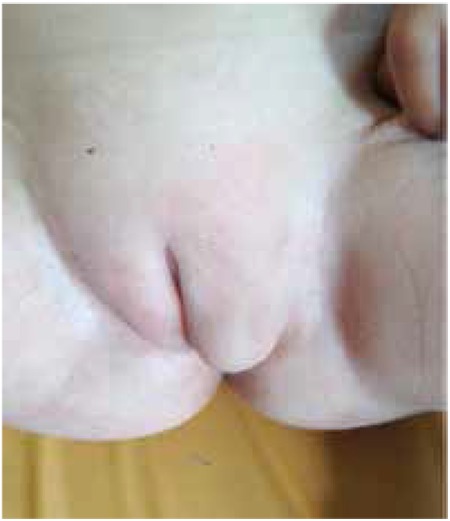
Female external genitalia and prolapse of the left labium majus due to the presence of a gonad (patient 2)

A 15-month-old patient was referred to the pediatric endocrinology outpatient clinic due to a mass in the inguinal region. Her parents were third-degree cousins. She was 77 cm in length and weighed 9650 g. There was a palpable mass in the right inguinal canal and a gonad was observed in the left labium majus. Cliteromegaly was not detected (Figure 1). Internal genitalia were consistent with male structures in ultrasonography. Laboratory results were as follows: FSH 1.25 IU/L, LH 0.00 IU/L, and T <200 pg/mL. An human chorionic gonadotropin (hCG) test was not performed. Karyotype was 47, and both XXY and SRY were positive. A V89L polymorphism and a homozygous mutation of p.Ala65Pro (c193G>C) nucleotide substitution in exon 1 of SRD5A2 gene were detected.

#### Patients 3, 4, 5, 6, Family 3

Patients 3, 4, 5, and 6 were all members of the same family. Patient 3 was a nine-year-old who presented with palpable bilateral masses in the inguinal area. Her parents were first-degree cousins. The patient was 137 cm [0.48 standard deviation score (SDS)] in height and 32.5 kg (0.45 SDS) in weight. Clitoromegaly was not detected. Hormone levels were as follows: FSH 1.57 IU/L, LH 0.15 IU/L, and T 110 pg/mL. After hCG simulation, was 1800 pg/mL, DHT was 53 pg/mL, and the T/DHT ratio was 33.9. As the patient’s all 3 siblings had histories of DSD, they were also evaluated within the scope of the study.

Patient 4 was 3 years old. Her height was 93 cm (-0.31 SDS) and weight was 15 kg (0.59 SDS). Gonads were palpable in both inguinal areas. After hCG simulation, T was 1020 pg/mL, DHT <20 pg/mL, and T/DHT ratio was >51.

Patient 5 was a 15-year-old and was admitted to the clinic with complaints of primary amenorrhea and masses in the inguinal region. She was 163 cm (0.24 SDS) tall and weighed 54.1 kg (0.3 SDS). Gonads were palpable bilaterally in the inguinal region. Breast development was at stage 1 and pubic hair growth was at stage 4 according to Tanner’s classification. Laboratory results were as follows: FSH 4.59 IU/L, LH 2.89 IU/L, T 1740 pg/mL, DHT 35 pg/mL, and the T/DHT ratio was 49.7.

Patient 6 was a 24-year-old with a history of inguinal hernia repair at the age of 10. Additionally, she was being followed-up for DSD and receiving hormone replacement therapy. Her height was 174.6 cm (2.9 SDS) and weight was 63.3 kg (1.8 SDS). Her thelarche was stage 4. The T/DHT ratio could not be analyzed in this patient.

**Figure 2:**
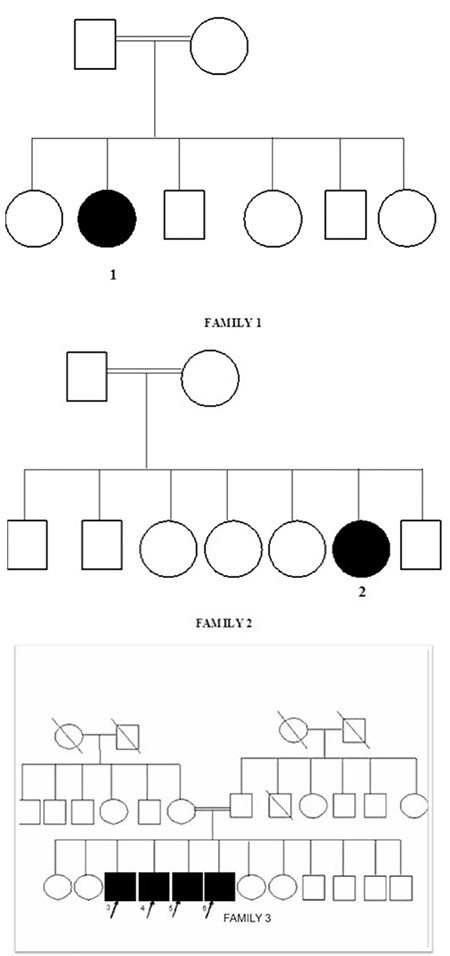
Pedigree of probands in Families 1, 2, 3: It was known that there was 5αRD2 deficiency in all patients

Uterus and ovary were not present in three patients: patients 3, 4, and 5. Their karyotype was 46,XY. Homozygous mutation of p.Ala65Pro (c193G>C) nucleotide substitution and V89L polymorphism in exon 1 of the SRD5A2 gene were detected. Figure 2 shows the pedigree of these probands.

### Genetic Analysis

**Figure 3:**
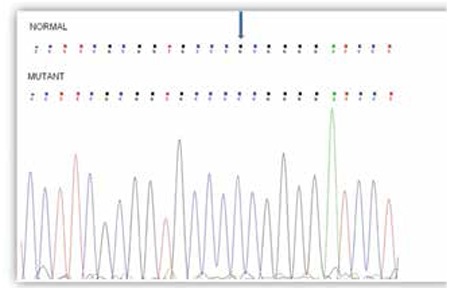
Mutation analyses of patients’ 5RD genes c.193G>C nucleotide substitution in exon 1

The six patients from Family 1, 2, and 3 were examined at Harran University Faculty of Medicine Hospital. Detailed clinical findings are presented in Table 1. Informed consent was obtained from the parents of all patients. Blood samples from the five patients were available for genetic analysis, and DNA was extracted from whole blood using a salting out procedure. Primers were designed for polymerase chain reaction amplification of five exons of the SRD5A2 gene (Table 2) (4), and the amplification products were sequenced on an Applied Biosystems 3730xl automated sequencer. The SRD5A2 gene sequence analyses of Patients 1-6 are shown in Figure 3. Based on the sequencing results, the SRD5A2 gene c.193G>C (p.Ala65Pro) nucleotide substitution in exon 1 was determined. This nucleotide variation identified at position 193 has caused the alteration of amino acid (Alanin-Prolin) in codon 65. Also, we have observed the SRY gene in all patients. In addition, we have found p.Leu89Val polymorphisms in all patients with 5αRD2 deficiency.

### Hormonal Evaluation

In all patients, blood FSH, LH, and total testosterone levels were determined using the electrochemiluminescence immunometric assay method with the Roche Elecsys E170 immuno-analyzer (Roche Diagnostics, Burgess Hill, UK). Serum DHT levels were measured with RIA.

## DISCUSSION

In humans, the SRD5A2 gene is located on chromosome 2p23 and contains 5 exons and 4 introns. 5αRD2 deficiency was first described in 1974 and genetic mutations were identified for nearly 20 years after that (5,6,7). More than 50 mutations have been identified to date (8,9). It has been reported that the incidence of the syndrome is high in the Dominican Republic, in some regions of New Guinea, and in Turkey. These findings are possibly related with “founder effect” and consanguineous marriages (10). Detection of the same mutation in different families in Iran has also been explained by the “founder effect” (11). The mutation identified in this study was detected in two patients who were the subjects of another study. The first patient was nine years old and reared as a girl. She presented to the clinic with the complaint of masses in both inguinal canals. There was a first-degree consanguinity between her parents. Genetic analysis revealed a mutation resulting from proline-to-alanine substitutions in exon 1, codon 65 (12). The second patient was seven years old and reared as a girl. She presented to the clinic with bilateral inguinal masses as well. Her parents were third-degree cousins. Genetic analysis revealed the same pAla65Pro mutation as in the first patient (13). We also detected the same mutations in three unrelated families from the same ethnic group. This finding has given rise to the thought that the existence of a common ancestor or a founder effect may be responsible for the spread of that genetic abnormality. This mutation, as far as we know, has not been detected in other ethnic populations.

Some disorders such as prostate cancer or hypospadias may be related with SRD5A2 gene polymorphisms. In the SRD5A2 gene, the V and L polymorphisms have been associated with 5αRD2 activity; while the V allele is considered to be related to high activity, the L allele is related to low activity. It has been found that having the V allele of the SRD5A2 gene doubles the risk of prostate cancer development (14). Specifically, these polymorphisms have been reported to increase the risk of prostate cancer in an Ecuadorian population (15). However, a meta-analysis involving 45 studies and a total of 15 562 patients was presented in 2013 and it was reported that there is no correlation between V89L polymorphism and prostate cancer although the A49T polymorphism may play a role in the etiology of prostate cancer in the Caucasian race (8). Another study revealed a strong correlation between V89L polymorphism and breast cancer (9).

A negative correlation has been noted between the V89 allele and hypospadias, implying that having the V allele may protect against hypospadias (14). It has been indicated that the individuals with the LL genotype of SRD5A2 in India are at a 3.6 times higher risk for hypospadias development. Additionally, as the individuals at risk for hypospadias are generally from agricultural regions, the probability of a correlation between pesticide exposure and risk of hypospadias has been highlighted (16). Various publications have reported the presence of V89L polymorphism in patients with 5αRD2 deficiency. The first patient, who appeared in 2005, was an eight-year-old who was reared as a boy and had a heterozygous A207D mutation and V89L polymorphism (17). A multi-center international study published in 2011 analyzed 55 patients with 5αRD2 deficiency and found heterozygous mutations in 69.1%, compound heterozygous mutations in 25.5%, and compound heterozygous mutations characterized by V89L polymorphism in 5.4% (n=3) of the patients (18). In India, a patient with perineoscrotal hypospadias and micropenis was observed to have a novel heterozygous missense mutation Q56H and V89L homozygous polymorphism (19). Maimoun et al (20) reported three newborns diagnosed with DSD and new mutations. It is remarkable that one of those three newborns was a Turkish patient with a micropenis, hypospadias, and bifid scrotum who had an S12R mutation in exon 1 as well as V89L polymorphism. All our patients were detected to have this polymorphism. It may be inferred that the V89L polymorphism is particularly common in our country. However, this is the first study that indicates the association of this mutation with V89L polymorphism. Our results confirm that V89L polymorphism affects the development of external genitalia.

Klinefelter’s syndrome is considered to be the most common chromosomal abnormality among males. DSD is not very common in individuals with Klinefelter’s syndrome. However, it was reported in 1994 that in 22 patients with ambiguous genitalia one had a 47,XXY genotype and another one had 46,XX/47,XXY. Another study carried out on 30 patients with DSD in 2010 reported 4 patients as having a 45,X/47,XXY pattern (21,22). Akcay and Ulucan (23) performed genetic analyses in three unvirilized patients with the 47,XXY genotype and found a p.g196s homozygous mutation in the SRD5A2 gene in patient one, 23 repeat polymorphisms in exon 1 of the androgen receptor gene in patient two, and heterozygous p.f891l mutations in androgen receptors, along with repeat polymorphisms, in patient three. In the present study, however, we reported a novel mutation of the SRD5A2 gene in combination with p.Ala65Pro in unvirilized patients with 47,XXY genotype. It should be kept in mind that in rare cases, Klinefelter patients may have ambiguous genitalia.

As a concluding remark, we could state that p.Ala65Pro mutation in the SRD5A2 gene causes 5αRD2 deficiency, especially in Turkey. V89L polymorphism may also be an important factor in the development of external genitalia.

## Ethics

Ethics Committee Approval: The present study was approved by local ethic committee (Harran University Faculty of Medicine), Informed Consent: It was taken.

Peer-review: External peer-reviewed.

## Figures and Tables

**Table 1:**
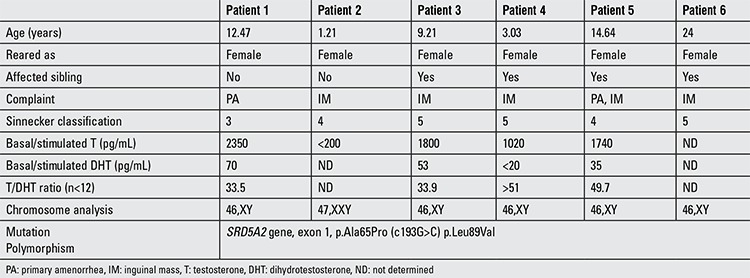
Clinical, laboratory, and genetic characteristics of the patients

**Table 2:**
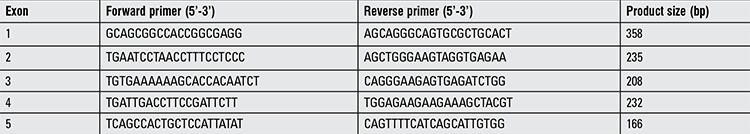
Primers and polymerase chain reaction products for exons and promoter regions of the SRD5A2 gene (23)
